# Outcomes of male patients with HR+/HER2– advanced breast cancer receiving palbociclib in the real-world POLARIS study

**DOI:** 10.1007/s10549-023-07145-1

**Published:** 2023-10-30

**Authors:** Joanne L. Blum, Caroline DiCristo, David Gordon, Meghan S. Karuturi, David Oubre, Erin Jepsen, Juan Cuevas, Shailendra Lakhanpal, Monica Z. Montelongo, Zhe Zhang, Joseph C. Cappelleri, Yao Wang, Debu Tripathy

**Affiliations:** 1 Baylor-Sammons Cancer Center, Texas Oncology, US Oncology, Dallas, TX USA; 2 Pfizer Inc, New York, NY USA; 3 Munson Medical Center, Traverse City, MI USA; 4 The University of Texas MD Anderson Cancer Center, Houston, TX USA; 5 Ponchartrain Cancer Center, Hammond, LA USA; 6 Novant Health Cancer Institute, Winston-Salem, NC USA; 7 St. Louis Cancer Care, St. Louis, MO USA; 8 Saint Vincent’s Birmingham, Birmingham, AL USA; 9 ICON plc, Blue Bell, PA USA; 10 Pfizer Inc, La Jolla, CA USA; 11 Pfizer Inc, Groton, CT USA

**Keywords:** Advanced breast cancer, Male, Hormone receptor–positive/human epidermal growth factor receptor 2–negative, Palbociclib, CDK4/6 inhibitor, Real-world

## Abstract

**Purpose:**

Data on treatments for male breast cancer patients are limited owing to rarity and underrepresentation in clinical trials. The real-world POLARIS study gathers data on palbociclib use for the treatment of hormone receptor–positive/human epidermal growth factor receptor 2–negative (HR+/HER2–) advanced breast cancer (ABC) in female and male patients. This sub-analysis describes real-world palbociclib treatment patterns, clinical outcomes, and quality of life (QoL) in male patients.

**Methods:**

POLARIS is a prospective, noninterventional, multicenter, real-world study of patients with HR+/HER2– ABC receiving palbociclib. Assessments included medical record reviews, patient QoL questionnaires (European Organisation for Research and Treatment of Cancer Quality-of-Life Questionnaire–Core 30), site characteristics questionnaires, and physician treatment selection surveys. Variables included demographics, disease history, global health status/QoL, clinical assessments and adverse events. Analyses were descriptive in nature. For clinical outcomes, real-world tumor responses and progression were determined by physician assessment in routine clinical practice. Real-world progression-free survival (rwPFS) was described using the Kaplan–Meier method.

**Results:**

At data cutoff, 15 male patients were enrolled (median age, 66 years). Nine patients received palbociclib as a first-line treatment and 6 as a second-line or later treatment. Patients received a median of 20 cycles of palbociclib. Neutropenia was experienced by 2 patients and grade ≥ 3 adverse events were reported in 11 patients. Global health status/QoL scores remained generally consistent during the study. One patient (6.7%) achieved a complete tumor response, 4 (26.7%) a partial response, and 8 (53.3%) stable disease. Median rwPFS was 19.8 months (95% CI, 7.4–38.0). Median follow-up duration was 24.7 months (95% CI, 20.0–35.7).

**Conclusion:**

This real-world analysis showed that palbociclib was well tolerated and provides preliminary data on treatment patterns and outcomes with palbociclib in male patients with HR+/HER2– ABC, helping inform the use of palbociclib in this patient subgroup.

**Trial identifier:**

NCT03280303.

**Supplementary Information:**

The online version contains supplementary material available at 10.1007/s10549-023-07145-1.

## Introduction

About 1% of all breast cancers occur in men [1]. In 2023, approximately 2800 new cases of male breast cancer are expected in the United States [1]. A nationwide, registry-based cohort study of patients diagnosed with primary breast cancer between 2004 and 2014 found male patients (*n* = 16,025) were typically older at diagnosis (mean [standard deviation] age, 63.3 [13.0] years) compared with female patients (*n* = 1,800,708; 59.9 [13.3] years) and more frequently had advanced disease (stage III, 14.0% vs 8.9%; stage IV, 5.8% vs 3.8%) [2]. Most breast cancer cases in the United States are hormone receptor–positive/human epidermal growth factor receptor 2–negative (HR+/HER2−), with higher percentages among male patients (78.3%–84.1%) versus female patients (66%–67.4%) [3–5].

Prognosis varies by cancer stage, with 5-year survival estimates for female breast cancer ranging from 98% for stage I to 27% for stage IV [4]. However, in the registry-based study mentioned previously, compared with female patients, male patients had significantly lower overall, 3-year, and 5-year survival rates; this disparity persisted across cancer stages, endocrine receptor and/or HER2 subtypes, age, years of diagnosis, and comorbidity status [2]. Male gender remained significantly associated with mortality in hazards regression models adjusted for age, clinical and treatment factors, race/ethnicity, and access to care. Another US study of 2010–2012 data identified worse overall survival among male (*n* = 1442) versus female (*n* = 172,847) breast cancer patients; survival differences were statistically significant for HR+ subtypes across several stages when adjusted for age, ethnicity, and tumor grade [3].

A more comprehensive understanding of the characteristics, treatments, and outcomes among male patients with breast cancer is needed, particularly in light of the exclusion of males from many breast cancer clinical trials [6]. Pivotal studies evaluating cyclin-dependent kinase 4/6 inhibitors (CDK4/6i) palbociclib, ribociclib, and abemaciclib, which are approved by the US Food and Drug Administration (FDA) in combination with the selective estrogen-receptor degrader fulvestrant or aromatase inhibitors (AIs) for treatment of HR+/HER2− metastatic or advanced breast cancer (MBC; ABC), did not provide data on male patients [7–9]. Of the seven registrational phase 3 CDK4/6i trials submitted to the FDA before January 2019, only one, MONALEESA-3, allowed male patients [10]; yet none were enrolled [11].

In 2019, the FDA expanded palbociclib indications to include male patients, based in part on real-world data from electronic health records and insurance claims from male patients with breast cancer treated with palbociclib (*N* = 12) [12], highlighting the potential role for real-world data in addressing unmet gaps in treatment. Recently, data from male breast cancer patients included in ribociclib and abemaciclib trials supported expanding approval of the CDK4/6i to include men [13–15]. The American Society of Clinical Oncology recommends CDK4/6i and other targeted therapies for MBC in male patients [16].

POLARIS is a real-world study investigating the use of palbociclib for the treatment of HR+/HER2− ABC in routine clinical practice [17]. Characteristics and treatment patterns among male patients included in POLARIS have been previously presented [18, 19]. This report includes updated data from male patients in POLARIS along with additional data, including patient-reported outcomes (PROs) and preliminary effectiveness estimates.

## Methods

### Study design and patients

**Fig. 1:**
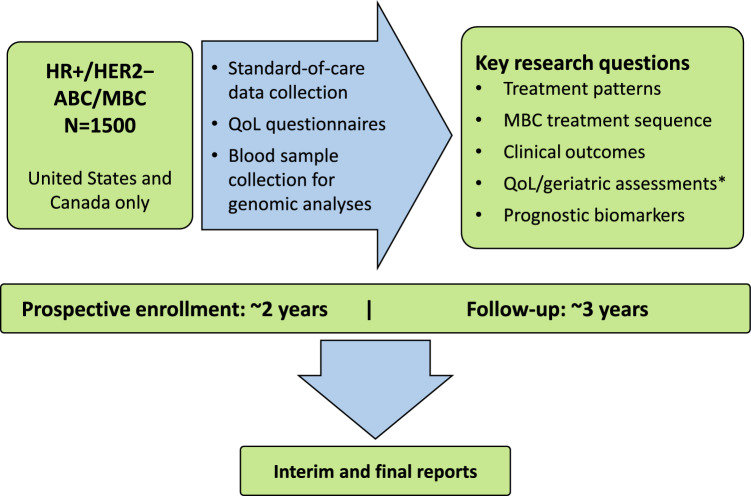
POLARIS study design. ABC, advanced breast cancer; HER2–, human epidermal growth factor receptor 2–negative; HR+, hormone receptor–positive; MBC, metastatic breast cancer; QoL, quality of life. *None of the male patients met criteria for collection of geriatric assessment

POLARIS is a prospective, noninterventional, multicenter study conducted in approximately 100 US and Canadian sites designed to provide real-world data on palbociclib use in patients with ABC (NCT03280303; Fig. 1). Enrollment of the targeted 1500 patients began on January 1, 2017, and closed September 30, 2019.

Eligible male patients were ≥ 18 years of age and diagnosed with HR+/HER2− carcinoma of the breast with evidence of advanced or metastatic disease not amenable to curative treatment and indicated by a physician for treatment with palbociclib. Key exclusion criteria were physician assessment of life expectancy < 3 months at the time of ABC diagnosis, participation in interventional clinical trials, and active treatment for malignancies other than ABC at enrollment.

The study was in accordance with legal and regulatory requirements. Prospective approvals of the study protocol and related documents were obtained from institutional review boards/independent ethics committees for each site. Patients provided written informed consent.

### Objectives and assessments

Primary objectives were to describe palbociclib prescribing and treatment patterns in routine clinical practice in ABC treatment, clinical outcomes, MBC treatment sequences, and patient quality of life (QoL).

The following data were collected: patient demographics, study site characteristics, medical history, concomitant medications, metastatic disease status, breast cancer diagnosis and recurrence history, breast cancer treatment, line of therapy, Eastern Cooperative Oncology Group (ECOG) performance status, clinical assessments, biomarker blood samples, complete blood count, comorbidities (per the Charlson Comorbidity Index), adverse events (AEs), and serious AEs. Line of therapy was defined as the number of therapies patients received after initial diagnosis of ABC up to and including palbociclib treatment start. Clinical and treatment data for each patient were derived from existing medical records. Treating physicians also completed a treatment selection survey at the start of palbociclib or any other therapy that captured the reason for treatment.

Patient global health status of prespecified patient-reported outcomes and overall QoL were assessed by the European Organisation for the Research and Treatment of Cancer Quality-of-Life Questionnaire–Core 30 (EORTC QLQ-C30), a cancer-specific 30-item questionnaire [20, 21], at baseline, monthly for the first three months of palbociclib treatment, and then every three months until the end of palbociclib treatment. Values from two items (overall health [item 29] and overall QoL [item 30]) on the EORTC QLQ-C30, each scored on a 7-point scale (not at all to very much), were averaged into one score to measure global health status/QoL; this score was transformed to a scale from 0 to 100; higher scores represent a higher QoL. Descriptive results were reported at a time point when an eligible patient responded to at least half of the items [21].

### Statistical analyses

Analyses were generally descriptive. Real-world tumor response (real-world complete response [rwCR], real-world partial response [rwPR], real-world stable disease [rwSD], and real-world progressive disease [rwPD]) was determined by physician assessment based on imaging, biopsies, biomarkers, and/or clinical judgment. Real-world response rate (rwRR) was the proportion of patients with a best tumor response of rwCR or rwPR during index treatment. Real-world clinical benefit rate (rwCBR) was calculated as the percentage of patients with best response of rwCR or rwPR at any time, or rwSD for ≥ 24 weeks from index treatment start date until first physician-documented rwPD during index treatment. Real-world progression-free survival (rwPFS) was defined as time (months) from palbociclib index treatment start date to the date of physician-documented progression or death due to any cause, whichever occurred first. Patients not experiencing the above events (i.e., death or progression) were censored at the date of last response assessment during index treatment; rwPFS was described using the Kaplan–Meier method. Duration of follow-up was calculated using the reverse Kaplan–Meier method.

## Results

### Patients

At data cutoff (August 26, 2021), there were 15 male patients among 1242 total patients who were enrolled and received at least one dose of study medication in POLARIS across 12 US sites. Patient demographic and baseline clinical characteristics are provided in Table 1. Median age at enrollment was 66 years (range, 43–82 years); nine patients were ≥ 65 years of age. Median age among patients receiving palbociclib therapy as first-line (1L), or second-line or later (2L+), was 70 years (range, 43–82 years) and 63.5 years (range, 52–69 years), respectively. Almost all patients were White (93.3%). Fourteen patients (93.3%) had ≥ 1 comorbidity, and two had ≥ 5 comorbidities at enrollment, and other than neoplasms, the most common comorbidities were vascular disorders (Supplementary Table 1). Concomitant medications received by ≥ 2 patients are listed in Supplementary Table 2.

**Table 1:** Baseline POLARIS patient demographic and clinical characteristics^a^

Characteristic	Male patients (*N* = 15)	Female patients (*N* = 1227)
Age distribution, y		
Median (range)	66 (43–82)	64 (22–97)
< 40	–	61 (5.0)
40–50	1 (6.7)	144 (11.7)
51– 69	9 (60.0)	614 (50.0)
70–74	4 (26.7)	177 (14.4)
75–84	1 (6.7)	197 (16.1)
85+	–	134 (2.8)
Race		
American Indian or Alaska Native	–	8 (0.7)
Asian	–	19 (1.5)
Native Hawaiian or Pacific Islander	–	5 (0.4)
White	14 (93.3)	1,005 (81.9)
Black	1 (6.7)	137 (11.2)
Other	–	23 (1.9)
Not reported	–	30 (2.4)
Ethnicity		
Hispanic/Latino	–	104 (8.5)
Not Hispanic/Latino	15 (100)	1,086 (88.5)
Not reported	–	36 (2.9)
ECOG performance status score		
0	7 (46.7)	489 (39.9)
1	5 (33.3)	455 (37.1)
2	2 (13.3)	88 (7.2)
3	–	20 (1.6)
Unknown	1 (6.7)	175 (14.3)
Stage of current diagnosis		
Locally advanced	1 (6.7)	62 (5.1)
Metastatic	14 (93.3)	1,162 (94.7)
Not reported	–	3 (0.2)
Disposition of diagnosis^b^		
Recurrent from earlier stage; stages 0–III	9 (60.0)	833 (67.9)
De novo; newly diagnosed stage IV at enrollment	6 (40.0)	333 (27.1)
Not reported	–	61 (5.0)
Distant metastases sites at MBC diagnosis^c^		
Median (range)	2.5 (1–5)	2.0 (1–10)
Adrenal gland	–	3 (0.3)
Bone	7 (50.0)	648 (55.8)
Brain	–	5 (0.4)
Chest wall	1 (7.1)	53 (4.6)
Liver	–	108 (9.3)
Lung	2 (14.3)	114 (9.8)
Lymph nodes—non-regional	–	22 (1.9)
Lymph nodes—regional	2 (14.3)	87 (7.5)
Other	1 (7.1)	46 (4.0)
Peritoneal	–	3 (0.3)
Pleura	–	31 (2.7)
Skin	1 (7.1)	28 (2.4)
Soft tissue	–	13 (1.1)
Visceral disease^d^		
Yes	7 (46.7)	478 (39.0)
No	8 (53.3)	749 (61.0)

ECOG, Eastern Cooperative Oncology Group; MBC, metastatic breast cancer

^a^All data are n (%) unless otherwise noted

^b^Patients with an initial stage of 0, I, or II are considered as having recurrent disease from an earlier stage; all other patients with non‑missing stage are de novo

^c^Among patients with metastatic disease only (male, *n* = 14; female, *n* = 1,161); multiple responses were allowed

^d^Visceral disease refers to metastases of the brain, liver, and/or lung/pleura at study enrollment

At enrollment, 14 patients (93.3%) had MBC; 6 (40.0%) had de novo metastatic disease (Table 1). Among patients with MBC, median number of metastatic sites was 2.5 (range, 1–5), the most common being bone (*n* = 7; 50.0%), followed by lungs and regional lymph nodes (*n* = 2 each; 14.3%). Seven patients (46.7%) had visceral disease at enrollment, defined as metastases of the brain, liver, and/or lung/pleura. Three patients (20.0%) had disease-free intervals (DFIs; time from date of first breast cancer diagnosis to onset of relapse/recurrent disease) of < 24 months, and five (33.3%) had DFIs of > 36 months. Demographic and clinical characteristics of the male patients were similar to those of the female POLARIS patients (Table 1).

### Treatment patterns

Nine patients (60.0%) initiated palbociclib as 1L treatment, the remainder initiating as 2L+ treatment. Among patients initiating 1L palbociclib, four (44.4%) received fulvestrant and five (55.6%) AIs (ie, letrozole or anastrozole) as the hormonal partner. Of the four patients who received fulvestrant, two had previously received tamoxifen and one received tamoxifen and anastrozole as adjuvant therapy. Of the five patients who received an AI, one received anastrozole and one received tamoxifen and letrozole as adjuvant therapy. Among those initiating 2L+ palbociclib, four (66.7%) and two (33.3%) received fulvestrant or AIs, respectively, as the hormonal partner.

Overall palbociclib prescribing and treatment patterns are described in Table 2. In seven patients (50.0%), palbociclib was selected owing to patient characteristics; primary reasons for other patients included likelihood of benefit (*n* = 5; 35.7%) and side-effect profile (*n* = 2; 14.3%). At data cutoff, patients had received a median of 20 palbociclib cycles (range, 2–41) lasting a median of 19.8 months (range, 1.4–38.1). Three patients (20.0%) had completed 24 months of treatment at the most recent follow-up, one (6.7%) completed 36 months of treatment, twelve (80.0%) discontinued palbociclib for reasons including disease progression (*n* = 8; 53.3%), toxicities/side effects (*n* = 2; 13.3%), or other (*n* = 2; 13.3%); treatment was ongoing for three patients (20.0%). Six patients (40.0%) were withdrawn during the study owing to death (*n* = 4; 26.7%) or other reasons (*n* = 2; 13.3% [1 withdrew consent; 1 no longer treated at study site]). Prescribing and treatment patterns of male patients were similar to those of the female POLARIS patients (Table 2).

**Table 2:** Palbociclib prescribing and treatment^a^

Characteristic	Male patients^b^	Female patients
Primary reason for selecting palbociclib		
Patient characteristics	7 (50.0)	626 (51.1)
Likelihood of clinical benefit	5 (35.7)	454 (37.0)
Side effect profile	2 (14.3)	70 (5.7)
Other	–	62 (5.1)
Cycles of palbociclib received		
Mean (SD)	16.9 (11.0)	15.7 (12.8)
Median (range)	20.0 (2–41)	12.0 (1–59)
Distribution		
1	0	78 (6.4)
2–4	2 (13.3)	220 (17.9)
5–9	3 (20.0)	231 (18.8)
10–14	2 (13.3)	139 (11.3)
≥ 15	8 (53.3)	559 (45.6)
Length of palbociclib treatment, mo		
Mean (SD)	16.6 (10.5)	15.0 (12.3)
Median (range)	19.8 (1.4–38.1)	11.8 (0–53.7)
Treatment disposition at most recent follow-up		
Treated for 24 months	3 (20.0)	292 (23.8)
Discontinued therapy	12 (80.0)	870 (70.9)
Disease progression	8 (53.3)	541 (44.1)
Toxicities or side effects	2 (13.3)	89 (7.3)
Other	2 (13.3)	113 (9.2)
Still receiving therapy	3 (20.0)	357 (29.1)
Withdrawn during the study	6 (40.0)	622 (50.7)
Patient died	4 (26.7)	385 (31.4)
Other	–	111 (9.0)
Adverse event	–	11 (0.9)
Lost to follow-up	1 (6.7)	24 (2.0)
No longer willing to participate in the study	1 (6.7)	91 (7.4)

SD, standard deviation

^a^All data are n (%) unless otherwise noted

^b^Denominator is *n* = 15 unless otherwise noted

Most patients initiated palbociclib treatment at 125 mg (Supplementary Table 3). Two patients (13.3%; both receiving 1L palbociclib [one with an AI, one with fulvestrant]) started cycle 1 at 100 mg because of comorbidities. Reasons for starting subsequent cycles at < 125 mg included comorbidities or previous treatment with reduced dose. Within the first 6 treatment cycles, three patients (cycle 1, n = 1 [6.7%]; cycle 2, *n* = 2 [13.3%]) had dosing interruptions based on patient decision, toxicities, or other reasons (*n* = 1 each), and one (7.1%; cycle 3) had a dose reduction to 75 mg for other reasons; no other dose modifications were reported during this period. Three of nine patients who initiated 1L palbociclib and one of six who initiated 2L+ palbociclib discontinued treatment within the first 6 cycles.

### Safety

Thirteen patients (86.7%) had AEs reported by the physician. The most common system organ classes to which AEs belonged were metabolism and nutrition disorders (*n* = 8 [53.3%]); respiratory, thoracic, and mediastinal disorders (*n* = 8 [53.3%]); blood and lymphatic system disorders (*n* = 7 [46.7%]); gastrointestinal disorders (*n* = 7 [46.7%]); and nervous system disorders (*n* = 5 [33%]). AEs reported by ≥ 3 patients included anemia, fatigue (*n* = 5 [33.3%] each), hypocalcemia, cough, diarrhea, nausea, peripheral edema, decreased neutrophil count, decreased white blood cell count, and peripheral neuropathy (*n* = 3 [20.0%] each). Two patients (13.3%) experienced neutropenia. Grade ≥ 3 AEs were experienced by 11 patients (73.3%); each grade ≥ 3 AE occurred in a single patient, except anemia and decreased white blood cell count (*n* = 2 [13.3%] each). One patient receiving palbociclib plus AI and one receiving palbociclib plus fulvestrant discontinued palbociclib owing to toxicity.

### Global health status/QoL

**Fig. 2:**
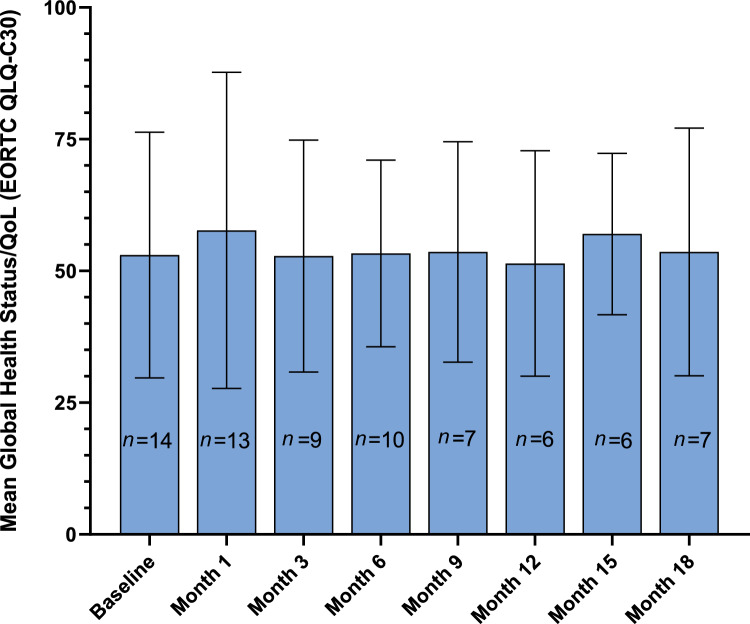
Global health status/quality of life (EORTC QLQ-C30) mean scores at different study time points. Error bars represent the standard deviations. EORTC QLQ-C30, European Organisation for the Research and Treatment of Cancer Quality-of-Life Questionnaire–Core 30; QoL, quality of life

Overall, for the first 18 months, global health status/QoL scores remained generally consistent among patients who responded (Fig. 2), although missing values generally increased over time. Mean scores were 53.0 at baseline and 57.7, 52.8, 53.3, 53.6, 51.4, 57.0, and 53.6 at months 1, 3, 6, 9, 12, 15, and 18, respectively.

### Clinical outcomes

Real-world best overall responses are listed by line of therapy in Table 3. Among all male patients, one (6.7%), treated with 1L palbociclib plus AI, experienced rwCR. Four patients (26.7%) experienced rwPR; two received palbociclib plus fulvestrant as 1L and two as 2L+. The rwRR for all patients was 33.3% (95% exact CI, 11.8–61.6) and was the same in subgroups analyzed by line of therapy. The overall rwCBR was 66.7% (95% exact CI, 38.4–88.2) and, among patients receiving 1L and 2L+ palbociclib, 55.6% and 83.3%, respectively.

**Table 3:** Real-world tumor responses and progression-free survival in POLARIS patients^a^

Response	All male patients (*N* = 15)	Male patients 1L treatment (*n* = 9)	Male patients 2L+ treatment (*n* = 6)	All female patients	Female patients 1L treatment	Female patients 2L+ treatment
Real-world tumor response				(*N* = 1226)^b^	(*n* = 895)	(*n* = 331)^b^
Best response						
Complete response	1 (6.7)	1 (11.1)	0	61 (5.0)	48 (5.4)	13 (3.9)
Partial response	4 (26.7)	2 (22.2)	2 (33.3)	300 (24.5)	245 (27.4)	55 (16.6)
Stable disease	8 (53.3)	4 (44.4)	4 (66.7)	488 (39.8)	343 (38.3)	145 (43.8)
Progressive disease	1 (6.7)	1 (11.1)	0	217 (17.7)	138 (15.4)	79 (23.9)
Indeterminate	1 (6.7)	1 (11.1)	0	160 (13.1)	121 (13.5)	39 (11.8)
Real-world response rate,^c^ *n* (%; 95% exact CI)	5 (33.3; 11.8–61.6)	3 (33.3; 7.5–70.1)	2 (33.3; 4.3–77.7)	361 (29.4; 26.9–32.1)	293 (32.7; 29.7–35.9)	68 (20.5; 16.3–25.3)
Clinical benefit rate,^d^ *n* (%; 95% exact CI)	10 (66.7; 38.4–88.2)	5 (55.6; 21.2–86.3)	5 (83.3; 35.9–99.6)	795 (64.8; 62.1, 67.5)	607 (67.8; 64.6–70.9)	188 (56.8; 51.3–62.2)
Real-world progression-free survival				(*N* = 1227)^b^	(*n* = 895)	(*n* = 332)^b^
Event	9 (60.0)	5 (55.6)	4 (66.7)	625 (50.9)	422 (47.2)	203 (61.1)
Death^e^	1 (11.1)	0	1 (25.0)	88 (14.1)	57 (13.5)	31 (15.3)
Progression^f^	8 (88.9)	5 (100.0)	3 (75.0)	537 (85.9)	365 (86.5)	172 (84.7)
Censored^g^	6 (40.0)	4 (44.4)	2 (33.3)	602 (49.1)	473 (52.8)	129 (38.9)
Kaplan–Meier estimate, median (95% CI), mo	19.8 (7.4–38.0)	21.8 (4.8–38.0)	14.8 (5.7–NE)	18.5 (17.1–20.3)	20.9 (18.5–24.2)	13.1 (10.6–16.4)

1L, first line; 2L, second line; CI, confidence interval; NE, not estimable

^a^All data are n (%) unless otherwise noted. Rates during index treatment with a data cutoff of August 26, 2021

^b^1226 total and 331 2L+ patients, respectively, were available for real-world tumor response analysis

^c^Percentage of patients with real-world best response of complete or partial response

^d^Percentage of patients with real-world best response of complete or partial response at any time or stable disease for ≥ 24 weeks from the index treatment start date

^e^Death without disease progression

^f^Physician documented disease progression

^g^Patients without events (death or progression) were censored at the last response assessment date during index treatment

**Fig. 3:**
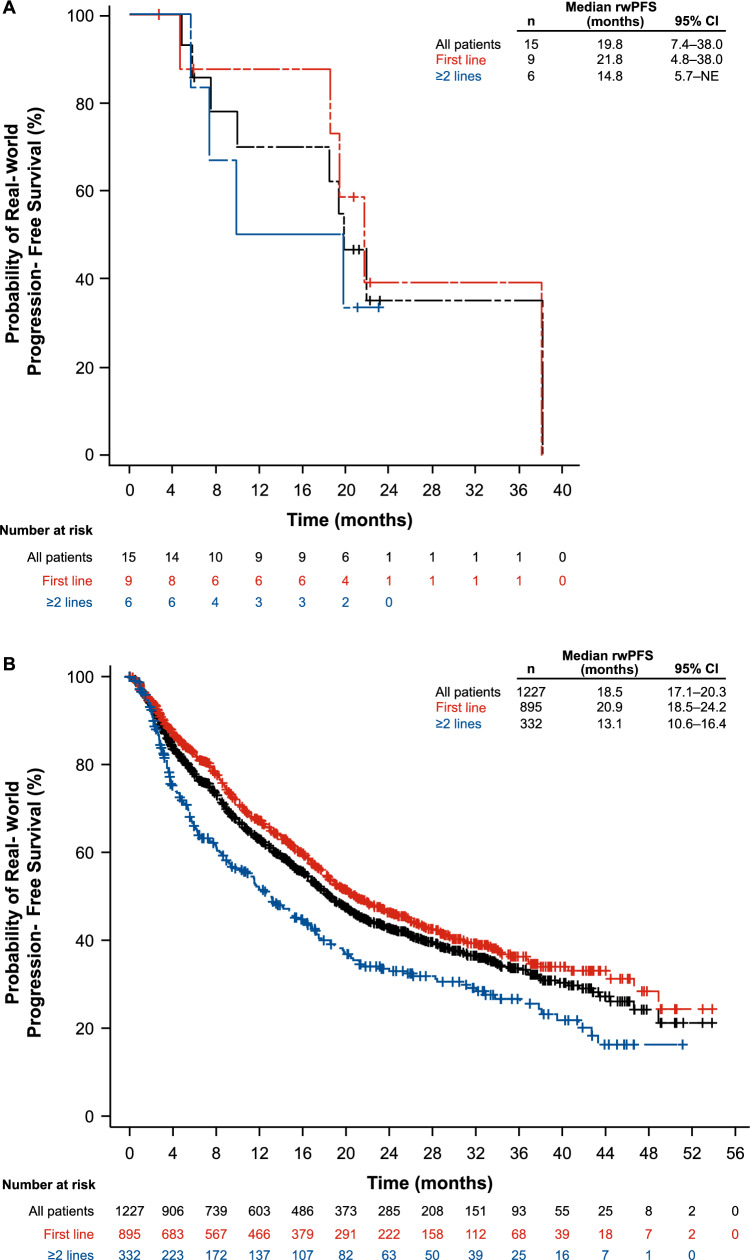
Kaplan–Meier curves showing real-world progression-free survival probability of male (**A**) and female (**B**) POLARIS patients. CI, confidence interval; NE, not estimable; rwPFS, real-world progression-free survival

Median follow-up for the male patients was 24.7 months (95% CI, 20.0–35.7). Median rwPFS was 19.8 months (95% CI, 7.4–38.0; Table 3 and Fig. 3A). When analyzed by palbociclib line of therapy, median rwPFS was 21.8 months (95% CI, 4.8–38.0) and 14.8 months (95% CI, 5.7-not estimable [NE]) in patients treated as 1L (2 de novo; 7 recurring disease, of which 4 had prior antiestrogen therapy) and 2L+ settings, respectively. rwPFS among female patients was similar (20.9 months [95% CI, 18.5–24.2] and 13.1 months [95% CI, 10.6–16.4]), respectively (Table 3 and Fig. 3B).

## Discussion

Considerable gaps remain in the understanding of male breast cancer treatment and outcomes, particularly because clinical trials generally exclude male patients [6]. This sub-analysis of men from the POLARIS study provides real-world evidence surrounding palbociclib treatment patterns, PROs, and clinical outcomes among US male patients with HR+/HER2− ABC. Treatment patterns, AEs, PROs, and outcomes among the men in this study were generally equivalent to those previously reported among predominantly female HR+/HER2− ABC patients treated with palbociclib.

In the female POLARIS population (*N* = 1227), 73% and 27% of patients received palbociclib plus endocrine therapy (ET) as 1L or 2L+ therapy, respectively (Table 3), versus 60% and 40% for the male patients in this study, respectively. Among the POLARIS patients receiving 1L treatment, rwRR and rwCBR were 32.7% and 67.8% (Table 3), and 33.3% and 55.6%, in female and male patients, respectively. Among patients receiving 2L+ treatment, rwRR and rwCBR were 20.5% and 56.8% (Table 3), and 33.3% and 83.3%, for female and male patients, respectively. Median rwPFS was 20.9 (95% CI, 18.5–24.2) and 13.1 (95% CI, 10.6–16.4) months (Table 3) for female patients receiving 1L or 2L+ treatment, respectively, versus 21.8 (95% CI, 4.8–38.0) and 14.8 (95% CI, 5.7-NE) months for males.

Baseline patient characteristics were consistent with those reported in other real-world studies. Median age at enrollment was 66 years, similar to mean ages reported for female patients in most real-world palbociclib studies (62.7–72 years) [22–26]. Percentages of patients with visceral metastasis (46.7%) and stage IV disease at initial diagnosis (40.0%) were also comparable to those reported in real-world palbociclib studies [22–27].

Treatment patterns in this study were consistent with those reported in the Ibrance Real-World Insights (IRIS) study among female patients with HR+/HER2− ABC/MBC [26]. In the IRIS study, 55.2% and 44.8% of patients received AI or fulvestrant, respectively, in combination with palbociclib. Among patients who received either palbociclib plus AI or palbociclib plus fulvestrant, 79.7% and 30.5%, respectively, received it as a 1L treatment, compared to 71.4% and 50.0% in the current study [26]. Other real-world studies in comparable female populations reported lower percentage of patients receiving palbociclib as 1L therapy, likely in part because the timing of palbociclib approval and, thus, may not have been available as 1L therapy for many patients [22, 25, 27]. The only study among these to provide insights on the choice of hormonal partners found that slightly more than half of patients (54.9%) received letrozole, 38.4% received fulvestrant, and 6.6% received other cancer therapies [27]. In terms of dosing, most patients in the current study initiated palbociclib at the recommended 125 mg dose, similar to other real-world studies, and dose modifications were relatively infrequent (6.7% in the current study vs 14.4%-33.7% in other reports) [22, 25–27].

Specific AEs reported for male patients in this study were comparable to those reported in clinical trials and real-world studies evaluating CDK4/6i use in combination with endocrine therapy among patients (almost exclusively female) with similar disease [11, 13, 14, 27–32], although the small number of patients in the current study may limit interpretation of the safety findings. One notable difference is the lower incidence of neutropenia (13.3%) reported in the current study, which is less than the 43.7% to 81% reported in clinical trials and among male patients in the CompLEEment-1 trial (53.8%) [14]. The rate of neutropenia was also lower than the 74.6% and 57.7% reported in two other real-world studies of female patients with ABC who received palbociclib in combination with various endocrine partners [25, 27]. Treatment discontinuation due to toxicities/side effects in the current study (13.3%) was somewhat higher than the 7.3% rate in the POLARIS female patients and the 7.6% rate reported among female patients in another real-world study [27] or the 4%-7.4% rates reported in palbociclib clinical trials [29, 30]; however, findings may not be comparable owing to variations in follow-up time and sample size, among other factors.

With consideration given to the small sample size in this study, global health status/QoL over 18 months remained similar to baseline. An earlier analysis of the broader POLARIS cohort (*n* = 522) had a higher baseline global health status/QoL (EORTC QLQ-C30) score of 66.2 compared with that of this male subset (53.0) but was likewise stable over the first 6 months of treatment [33]. Corresponding baseline scores in the phase 3 PALOMA-3 trial in female patients treated with palbociclib plus fulvestrant (mean score, 65.9) were similar to those in the broader POLARIS study and also showed minimal changes throughout the study (0.9-point decrease after a median of 5.6 months of follow-up) [34, 35].

In large clinical trials evaluating use of CDK4/6i in female patients with breast cancer as 1L or 2L+, response rates for palbociclib, ribociclib, and abemaciclib were 42.1%, 42.5%, and 49.7%, respectively, when coadministered with an AI, and 25.0%, 32.4%, and 35.2% when coadministered with fulvestrant [11, 28–32]. In the recent CompLEEment-1 trial, which evaluated ribociclib plus letrozole use in patients with HR+/HER2− ABC, male patients (*n* = 39) had a response rate (46.9%) similar to all patients (*n* = 2079; 43.6%) [14]. An analysis of 12 male MBC patients receiving palbociclib across all lines of therapy from the Flatiron Health database found that 33% achieved a rwRR (2 rwCR and 2 rwPR) [36]. Although the 33.3% rwRR reported for men receiving 1L palbociclib in the current study is similar to this rwRR in male patients from the Flatiron Health database and the rwRR of 32.7% in the 1L female POLARIS patients, it is numerically lower than that reported in other real-world studies in female patients with HR+/HER2− MBC treated with 1L palbociclib plus letrozole (adjusted rates, 58.6%-59.3%) [23] or with HR+/HER2− ABC/MBC treated with 1L palbociclib plus AI (79.5%) or 1L palbociclib plus fulvestrant (69.2%) [26]. Importantly, in vitro, palbociclib is cytostatic [7]; therefore, the high percentage of patients experiencing stable disease in this study (53.3%), particularly in the 2L+ setting, likely reflects clinical benefit observed in most or all patients.

In contrast to the rwRR, median rwPFS reported in the current study (1L, 21.8 months; 2L+, 14.8 months) was comparable to the female POLARIS patients as well as to that reported in two recent real-world studies conducted in a comparable female population, and predominantly female population, receiving first-line palbociclib plus letrozole, where adjusted median rwPFS were 20.0 months and 19.3 months, respectively [24, 37], but higher than rwPFS from another real-world study in which female patients received either 1L/2L+ palbociclib plus letrozole or palbociclib plus fulvestrant (median rwPFS, 8.9 months and 10.3 months, respectively) [27]. Median PFS estimates were 24.8, 25.3, and 28.2 months in clinical trials investigating CDK4/6i in combination with an AI and 9.5, 20.5, and 16.9 months in combination with fulvestrant [11, 28–30, 32, 38]. A pooled analysis of these trials estimated median PFS of 28.0 months when a CDK4/6i was administered with an AI in the 1L setting [10]. In addition, a recent real-world comparative effectiveness study of patients with HR+/HER2− ABC from the Flatiron Health database demonstrated significantly prolonged overall survival in the subgroup of male patients receiving palbociclib plus letrozole (*n* = 17) versus AI alone (*n* = 12), supporting the effectiveness of palbociclib in male patients [37].

Caution should be taken when comparing results from this study with those from clinical trials or other real-world studies; individual studies use different inclusion and exclusion criteria and, therefore, may not be comparable. Also, the sample size in the current study is very small and included patients who initiated palbociclib treatment with different endocrine partners and at any line of treatment, whereas other studies may be more restrictive with regard to these parameters. Tumor assessments in the real-world setting were not performed on a fixed schedule per Response Evaluation Criteria in Solid Tumors (RECIST) criteria, limiting comparison to clinical trials. Real-world studies by design are inherently subject to selection bias and missing information for some patients, which limits generalizability and comparison to clinical trials.

## Conclusions

Few studies have evaluated real-world patient characteristics and outcomes in male patients with HR+/HER2− ABC. In this prospective real-world study of male patients with HR+/HER2− ABC, safety analyses indicated that palbociclib was well tolerated, with an AE profile equivalent to that reported for female patients in real-world studies. This analysis also contributes to the understanding of differences between male and female patients with HR+/HER2– breast cancer regarding treatment patterns and outcomes, which is critical given tumor biology may differ between these populations. Although further studies in male patients are needed to fully understand potential differences in outcomes and safety versus female patients, this study provides additional data that can supplement registry or other case sources to better understand treatment strategies to optimize the benefit-risk profile and the impact of palbociclib (in combination with ET) in male patients with HR+/HER2− ABC.

### Supplementary Information

Below is the link to the electronic supplementary material.

Supplementary file1 (PDF 185 KB)

## Data Availability

Upon request, and subject to review, Pfizer will provide the data that support the findings of this study. Subject to certain criteria, conditions and exceptions, Pfizer may also provide access to the related individual de-identified participant data. See https://www.pfizer.com/science/clinical-trials/trial-data-and-results for more information.
