# Supplementary material for: Outcomes of male patients with HR+/HER2– advanced breast cancer receiving palbociclib in the real-world POLARIS study
Source: Breast Cancer Res Treat. 2023 Oct 30;203(3):463–75. doi: 10.1007/s10549-023-07145-1 (PMC10805882; doi:10.1007/s10549-023-07145-1)
Supplement: Supplementary file 1 — Supplementary file1 (PDF 185 KB) [file 10549_2023_7145_MOESM1_ESM.pdf]

Supplementary Material

Outcomes of Male Patients with HR+/HER2– Advanced Breast Cancer Receiving Palbociclib in the Real-World POLARIS Study

Joanne L. Blum, Caroline DiCristo, David Gordon, Meghan S. Karuturi, David Oubre, Erin Jepsen, Juan Cuevas, Shailendra Lakhanpal, Monica Z. Montelongo, Zhe Zhang, Joseph C. Cappelleri, Yao Wang, Debu Tripathy

Breast Cancer Research and Treatment

Corresponding Author:
Joanne L. Blum
Baylor-Sammons Cancer Center, Texas Oncology, US Oncology
Dallas, TX, USA
jlblum@att.net

Supplementary Table 1. Comorbidities occurring in patients

Comorbidity,[a] n (%)	Overall Cohort (N=15)
Total number of comorbidities	
0	1 (6.7)
1–2	6 (40.0)
3–4	6 (40.0)
≥5	2 (13.3)
Frequency of comorbidities[b]	
Blood and lymphatic system disorders	3 (20.0)
Cardiac disorders	1 (6.7)
Infections and infestations	1 (6.7)
Metabolism and nutrition disorders	5 (33.3)
Neoplasms benign, malignant, and unspecified (including cysts and polyps)	12 (80.0)
Psychiatric disorders	4 (26.7)
Renal and urinary disorders	3 (20.0)
Respiratory, thoracic, and mediastinal disorders	2 (13.3)
Vascular disorders	8 (53.3)

[a]Multiple responses allowed. Proportion of patients with each comorbidity reported only among patients with non-missing comorbidity data.
[b]Number of comorbidities calculated from among the comorbidities listed (ie,

Charlson Index comorbidities, plus hematologic and other comorbidities listed by the site investigators.)

Supplementary Table 2. Documented concomitant medications received by ≥2 patients

Medication, n (%)	Patients (N=15)
Analgesics	10 (66.7)
Paracetamol	3 (20.0)
Hydrocodone plus acetaminophen	2 (13.3)
Antihyperglycemics	4 (26.7)
Metformin	4 (26.7)
Glimepiride	2 (13.3)
Vitamins	8 (53.3)
Cholecalciferol	6 (40.0)
Antiemetics and antinauseants	5 (33.3)
Prochlorperazine	3 (20.0)
Ondansetron	2 (13.3)
Peptic ulcer and GERD medications	4 (26.7)
Pantoprazole	2 (13.3)
Psychoanaleptics	5 (33.3)
Psycholeptics	5 (33.3)
Alprazolam	4 (26.7)
Buspirone	2 (13.3)
Zolpidem	2 (13.3)
Antihyperlipidemics	4 (26.7)
Atorvastatin	2 (13.3)
Renin-angiotensin agents	6 (40.0)
Losartan	3 (20.0)
Lisinopril	2 (13.3)
Mineral supplements	4 (26.7)
Cardiac therapy	3 (20.0)
ACE inhibitors	2 (13.3)
Lisinopril	2 (13.3)
Diuretics	5 (33.3)
Furosemide	2 (13.3)
Hydrochlorothiazide	2 (13.3)
Antihypertensives	2 (13.3)
Beta-blocking agents	2 (13.3)
Obstructive airway disease agents	2 (13.3)

Nasal preparations	2 (13.3)
Antithrombotic agents	4 (26.7)
Rivaroxaban	2 (13.3)
Antianemic preparations	2 (13.3)
Cyanocobalamin	2 (13.3)
Urologicals[a]	2 (13.3)
Thyroid medications	3 (20.0)
Levothyroxine	3 (20.0)

ACE=angiotensin-converting enzyme; GERD=gastro-esophageal reflux disease.
[a]Genitourinary system medications.

Supplementary Table 3. Palbociclib prescribing and treatment during the first 6 treatment cycles[a]

Treatment Cycle<br>Characteristic	Patients
**Palbociclib cycle 1**	
Patients, n	15
Palbociclib starting dose, mg	
125	13 (86.7)
100	2 (13.3)
Palbociclib dose modification	1 (6.7)
Dosing Interruption[b]	1 (6.7)
Reason for dose modification[c]	
Patient decision	1 (100.0)
**Palbociclib cycle 2**	
Patients, n	15
Palbociclib starting dose, mg	
125	13 (86.7)
100	2 (13.3)
Palbociclib dose modification	2 (13.3)
Dosing Interruption[b]	2 (13.3)
Reason for dose modification[c]	
Toxicities or side effects	1 (50.0)
Other	1 (50.0)
**Palbociclib cycle 3**	
Patients, n	14
Palbociclib starting dose, mg	
125	13 (92.9)
100	1 (7.1)
Palbociclib dose modification	1 (7.1)
Decrease to 75 mg	1 (7.1)
**Palbociclib cycle 4**	
Patients, n	14
Palbociclib starting dose, mg	
125	12 (85.7)
100	1 (7.1)
75	1 (7.1)
Palbociclib dose modification	0

**Palbociclib cycle 5**	
Patients, n	13
Palbociclib starting dose, mg	
125	12 (92.3)
100	1 (7.7)
Palbociclib dose modification	0
**Palbociclib cycle 6**	
Patients, n	11
Palbociclib starting dose, mg	
125	10 (90.9)
100	1 (9.1)
Palbociclib dose modification	0

[a]All data are n (%) unless otherwise noted.

[b]No change in dose received after interruption.

[c]Denominator used is the number of patients for that cycle with a dose modification.
